# ASO Author Reflections: Increasing Age is Associated with Increased Risk of Long-Term Mortality After Surgery and Non-operation in Esophagogastric Cancer Patients

**DOI:** 10.1245/s10434-023-13205-z

**Published:** 2023-02-15

**Authors:** Joonas H. Kauppila, Fredrik Mattsson, Jesper Lagergren

**Affiliations:** 1 Upper Gastrointestinal Surgery, Department of Molecular Medicine and Surgery, Karolinska Institutet, and Karolinska University Hospital, Stockholm, Sweden; 2 Surgery Research Unit, Medical Research Center, University of Oulu and Oulu University Hospital, 90014 Oulu, Finland; 3 School of Cancer and Pharmaceutical Sciences, King’s College London, and Guy’s and St Thomas’ NHS Foundation Trust, London, UK

## Past

Esophageal and gastric cancers are among the most deadly cancers globally. The surgical treatment of esophagogastric cancers is extensive, with a high incidence of complications. The peak incidence of these cancers is at around 70–75 years of age. In previous studies, older age has been linked to increased mortality in surgically treated esophageal and gastric cancer;^1–4^ however, these studies categorized age in larger groups based often on single cut-offs. Therefore, it is relevant to assess age in relation to prognosis of surgically treated patients with more detailed analysis regarding which age these risks start to increase.

## Present

In our study,^5^ we sought to evaluate the association between age (in 5-year age groups) and prognosis of esophagogastric cancer patients undergoing surgery and the risk of non-operative treatment in a population-based nationwide cohort in Sweden. Among the 11,207 patients who underwent surgery, the risk for 5-year mortality was significantly higher in the 65–69 years age group compared with age <50 years, with increasing hazard with increasing age. Overall, in 28,725 esophagogastric patients, the odds of non-operation was significantly higher in the 75–79 years age group compared with age <50 years, with increasing odds with increasing age. We concluded that while increasing age is associated with a higher risk of mortality, the increasing odds of non-operation suggests a level of undertreatment depending on patient age.

## Future

As the populations in many countries get older, patient age will increasingly need to be considered in clinical decision making. Patients, especially those aged ≥65 years, should be part of the decision-making process regarding whether to undergo surgery or not. Older age is a risk factor for worse survival, but there might also be a level of undertreatment of patients due to age. Future studies should determine factors influencing survival in different age groups, e.g. objective assessment of fitness, such as exercise tests, stair-climbing tests, and spirometry. This would allow more robust evaluation of the potential benefits and harms that surgery may bring to different age groups of patients with esophagogastric cancer.
